# Effect of urea and squaramide IMPDH inhibitors on *C. parvum*: *in vitro* trial design impacts the assessment of drug efficacy

**DOI:** 10.1016/j.ijpddr.2025.100592

**Published:** 2025-04-15

**Authors:** Anne-Charlotte Lenière, Amit Upadhyay, Jérôme Follet, Timothy P. O'Sullivan

**Affiliations:** a University of Lille, CNRS, Centrale Lille, Junia, Université Polytechnique Hauts de France, UMR 8520, IEMN Institut d’Electronique de Microélectronique et de Nanotechnologie, F59000, Lille, France; b School of Chemistry, University College Cork, Cork, T12 YN60, Ireland; c School of Pharmacy, University College Cork, Cork, T12 YN60, Ireland; d Analytical and Biological Chemistry Research Facility, University College Cork, Cork, T12 YN60, Ireland

**Keywords:** Cryptosporidium parvum, Antiparasitic, IMPDH, Urea, Squaramide, Bioisostere, Heteroaryl

## Abstract

The protozoan parasite *Cryptosporidium* is the etiological agent of cryptosporidiosis, a ubiquitous diarrheic disease affecting humans and animals. Treatment options are limited, highlighting an urgent need for novel therapeutics. Despite decades of research and a wide diversity of strategies to tackle parasite metabolic pathways, no completely effective drug has been identified to date. Within targeted parasite enzymatic and metabolic pathways, the synthesis of nucleotide mediated by the inosine 5′-monophosphate dehydrogenase (IMPDH) enzyme is the focus of significant research efforts. Based on our prior studies of bacterial IMPDH inhibitors, we report herein the development and characterisation of novel inhibitors targeting *Cryptosporidium parvum* IMPDH (*Cp*IMPDH). Specifically, we synthesised heteroaryl-containing urea and squaramide analogues to evaluate their potential *in vitro* anti-*Cryptosporidium* activity. Initial screening identified nine active compounds with the most potent candidates achieving IC_50_ values as low as 2.2 μM. Subsequent time-course experiments revealed that the molecules effectively inhibit parasite invasion and early intracellular development but failed to tackle *C. parvum* growth when introduced at 30 h post infection. The present work introduces a new family of squaramide-derived IMPDH inhibitors and also interrogates the need to standardise commonly accepted protocols used for assessing anti-cryptosporidial drug activity.

## Highlights

• A series of urea and squaramide IMPDH inhibitors were evaluated against *C. parvum.*
• Several compounds displayed potent activity with IC_50_ values as low as 2.2 μM.
• Time-course experiments confirmed that the active compounds inhibit parasite invasion and early intracellular development.

## Graphical abstract

**Figure:**
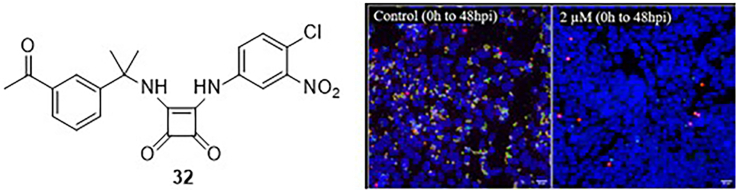

## Abbreviations

ATR: Attenuated Total Reflectance
*Cp*IMPDH: *C. parvum* IMPDH
dba: Dibenzylideneacetone
DBU: 1,8-Diazabicyclo(5.4.0)undec-7-ene
dGTP: 2′-Deoxyguanosine-5′-triphosphate
DMSO: Dimethyl sulfoxide
DNA: Deoxyribonucleic acid
GMP: Guanosine 5′-monophosphate
GTP: Guanosine 5′-triphosphate
HCT8: human intestinal epithelial cell line derived from colorectal adenocarcinoma
hpi: Hours post infection
HRMS: High-resolution mass spectrometry
IMP: Inosine 5′-monophosphate
IMPDH: Inosine 5′-monophosphate dehydrogenase
IR: Infra-red
MW: Microwave
NAD: Nicotinamide adenine dinucleotide
PI(4)K: Phosphatidylinositol 4-Kinase
Phe-RS: Phenylalanine tRNA synthetase
RNA: Ribonucleic acid
TBD: 1,5,7-Triazabicyclo[4.4.0]dec-5-ene
TMG: 1,1,3,3-Tetramethylguanidine
XMP: Xanthosine-5′-monophosphate

## 1 Introduction

*Cryptosporidium* spp. are zoonotic parasites responsible for cryptosporidiosis, a disease which causes moderate to severe diarrhea, nausea, abdominal pain and fever in a wide range of hosts, including humans (Innes et al., 2020). Among the 44 species and over 120 genotypes of *Cryptosporidium* identified (Ryan et al., 2021), *C. parvum* and *C. hominis* are responsible for more than 90 % of human cases (Chalmers et al., 2011). This parasite is transmitted from animals to humans during zoonotic episodes, most often by ingestion of parasites oocysts *via* the faecal-oral route. *Cryptosporidium* is one of the four main pathogens responsible for severe diarrhea, along with Rotavirus, *Shigella* and *Escherichia coli* (Kotloff et al., 2013). Globally, cryptosporidiosis poses a significant public health burden. It is estimated that 7.6 million cases of *Cryptosporidium* spp. infections occur annually, resulting in an estimated 202,000 deaths, predominantly among children under five in low-income regions (Liu et al., 2012; Striepen, 2013; Sow et al., 2016; Khalil et al., 2018). In addition, immunocompromised, elderly or malnourished people are highly susceptible to this parasite and can develop chronic forms of cryptosporidiosis, ultimately leading to death (Manabe et al., 1998; Checkley et al., 2015).

Despite the widespread prevalence of cryptosporidiosis, treatment options remain limited. A vaccine has been developed for cattle (Timmermans et al., 2024), but to date, no vaccine is available for humans. A wide range of enzymes and metabolic pathways have been investigated as promising targets in *in vitro and in vivo* trials (for a recent review see Lenière et al., 2024), but so far nitazoxanide is the only FDA-approved drug for cryptosporidiosis (Fox and Saravolatz, 2005). This compound inhibits the anaerobic metabolism of the parasite by targeting the pyruvate-ferredoxin oxidoreductase enzyme (Fox and Saravolatz, 2005). However, it is ineffective in immunocompromised patients, even when administered in high doses (Rossignol et al., 1998). Its efficacy in children is also limited (Amadi et al., 2002). As a consequence, it was not approved for use in children under one year. There is, therefore, an urgent need for the development of new therapeutic options against *Cryptosporidium* infections.

Inosine 5′-monophosphate dehydrogenase (IMPDH) has been the subject of increasing interest as a target for the development of novel antimicrobial agents (Cuny et al., 2017; Ayoub et al., 2024a). This enzyme has been characterised in a broad range of apicomplexan parasites such as *Plasmodium* (Raza et al., 2017), *Eimeria* (Hupe et al., 1986), *Toxoplasma* (Sullivan et al., 2005), *Babesia* (Cao et al., 2013) and *Cryptosporidium* (Hedstrom et al., 2011). IMPDH plays an important role in nucleotide biosynthesis where it catalyses the conversion of inosine-5′-monophosphate (IMP) into xanthosine-5′-monophosphate (XMP) using NAD + as a cofactor (Sintchak and Nimmesgern, 2000). XMP is subsequently transformed by GMP synthase into guanosine monophosphate (GMP) which serves as a critical precursor molecule for DNA (i.e. 2′-deoxyguanosine-5′-triphosphate dGTP) and RNA (i.e. guanosine-5′-triphosphate GTP) production. *C. parvum* is unable to synthesise purine nucleotides *de novo* and instead relies on host adenosine which is converted into guanine nucleotides under IMPDH catalysis (Abrahamsen et al., 2004). Genetic analysis suggests that the parasite likely obtained IMPDH *via* lateral gene transfer from an e-proteobacterium (Striepen et al., 2002). Although the same reactions are catalysed by both eukaryotic and prokaryotic IMPDH, the IMPDH enzymes in eukaryotes and prokaryotes are characterised by different structural and biochemical features (Hedstrom et al., 2011; Shah and Kharkar, 2015; Buey et al., 2022; Ayoub et al., 2024a). For example, while the IMP and nicotinamide binding sites are highly conserved, the adenosine and pyrophosphate sites are distinct (Makowska-Grzyska et al., 2015). As *Cp*IMPDH differs significantly from mammalian IMPDH, the parasite can be targeted without impacting on the host. Furthermore, compounds which have been found to inhibit bacterial IMPDH may also have application against *Cp*IMPDH.

Recent data have highlighted that IMPDH is not essential for parasite survival (Pawlowic et al., 2019) and suggested the existence of as yet undiscovered purine transporters or salvage enzymes. But the methodological approach based on IMPDH ablation may have artificially selected adapted parasites using a bypass metabolic pathway to circumvent the absence of IMPDH. In unmodified parasites, it could be suggested that the IMPDH pathway remains a vulnerability in parasite metabolism, particularly during the early life stages, prior to the selection processes induced by the absence or inhibition of IMPDH activity.

**Fig. 1:**
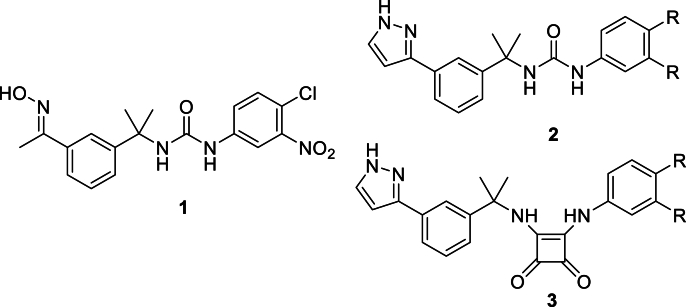
Lead oxime **1** compared to heteroaryl-containing ureas **2** and squaramides **3**.

We recently reported the discovery of a series of novel inhibitors of bacterial IMPDH (Ayoub et al., 2024b). These compounds were based on *Cp*IMPDH inhibitors originally reported by Hedstrom and colleagues (Gorla et al., 2012; Mandapati et al., 2014). Whereas lead compound **1** contained an oxime functional group which was prone to metabolic degradation, our compounds incorporated stable heteroaryl rings as in **2** (Fig. 1). These heteroaryl-containing analogues successfully inhibited IMPDH in *P. aeruginosa*, *S. aureus* and *E. coli* at submicromolar concentrations. However, they have not been previously evaluated against *C. parvum*.

The squaramide group has emerged as a possible bioisosteric replacement for ureas (Marchetti et al., 2019; Chasák et al., 2021). Given our previous success in replacing the oxime with a heteroaromatic ring, we wondered if incorporation of a squaramide into **3** in place of the urea group in **2** might also be worthwhile. Accordingly, in this paper, we outline our efforts in screening a library of heteroaryl-containing ureas for antiparasitic activity. We also describe the synthesis of a family of novel squaramide analogues and their subsequent *in vitro* evaluation against *C. parvum*.

## 2 Materials and methods

### 2.1 Synthesis

Acetonitrile, acetyl chloride, *n*-butyllithium, dichloromethane, dioxane, ethanol, ethyl acetate, hydrochloric acid, methanol, methyl magnesium bromide, sodium bicarbonate, sodium carbonate, sodium hydroxide, sodium azide, tetrahydrofuran, triphenylphosphine and tripotassium phosphate were obtained from Sigma-Aldrich (Gillingham, United Kingdom). Diethyl squarate, hexane, tetrakis(triphenylphosphine)palladium, tetramethyl guanidine, titanium isopropoxide, triethylamine and tris(dibenzylideneacetone)dipalladium were obtained from Fluorochem Ltd (Hadfield, United Kingdom). The boronic acids/esters were also obtained from Fluorochem Ltd (Hadfield, United Kingdom). Ethyl chloroformate and toluene were obtained from Thermo Fisher Scientific (Blanchardstown, Ireland). 4-Chloro-3-nitroaniline was obtained from TCI Chemicals (Zwijndrecht, Belgium). Unless otherwise noted, all the purchased materials and solvents were used without further purification. Compounds were purified by silica gel (Kieselgel 60, 0.040–0.063 mm, Merck) column chromatography. ^1^H NMR and ^13^C NMR spectra were recorded on Bruker Avance 300 (300/75 MHz), Bruker Avance 400 (400/100 MHz), Bruker Avance 500 (500/125 MHz) or Bruker Avance 600 (600/150 MHz) NMR spectrometers respectively.

#### 2.1.1 Synthesis of 1-(2-(3-bromophenyl)propan-2-yl)-3-(4-chloro-3-nitrophenyl)urea (**11**)

To a solution of **4** (2.00 g, 8.227 mmol, 1.0 eq.) in dichloromethane (20 ml), cooled to 0 °C, was added triethylamine (1.5 ml, 10.695 mmol, 1.3 eq.). Ethylchloroformate (0.9 ml, 9.872 mmol, 1.2 eq.) was added dropwise and the reaction mixture was stirred at the same temperature for 1 h. Sodium azide (588 mg, 9.050 mmol, 1.1 eq.) was added and the reaction mixture was stirred at room temperature for 18 h. After completion, the reaction mixture was filtered and the filtrate was concentrated under vacuum to afford 2-(3-bromophenyl)-2-methylpropanoyl azide as a brown coloured oil. The azide intermediate was then dissolved in toluene (15 ml) and was heated to reflux for 2 h. The reaction mixture was concentrated under vacuum to give 1-bromo-3-(2-isocyanatopropan-2-yl)benzene as a black coloured oil (1.9 g, 7.916 mmol). A solution of 1-bromo-3-(2-isocyanatopropan-2-yl)benzene (1.9 g, 7.916 mmol, 1.0 eq.) in tetrahydrofuran (20 ml) was cooled to 0 °C and triethylamine (1.2 ml, 8.708 mmol, 1.1 eq.) was added. After stirring the reaction mixture for 10 min, 4-chloro-3-nitroaniline (1.63 g, 9.500 mmol, 1.2 eq.) was added and the reaction mixture was stirred at room temperature for 18 h. After completion, the reaction mixture was diluted with water (20 ml) and extracted with ethyl acetate (2 × 25 ml). The combined organic layer was washed with brine (1 × 20 ml) and dried over magnesium sulfate. The organic layer was concentrated under vacuum to give a residue which was purified by silica gel column chromatography in ethyl acetate-hexane (0 %–30 %) to afford arylbromide **11** as an off-white solid (2.10 g, 5.088 mmol, 62 %).

#### 2.1.2 Synthesis of 1-(4-chloro-3-nitrophenyl)-3-(2-(3′-nitro-[1,1′-biphenyl]-3-yl)propan-2-yl)urea (**12**)

A solution of **11** (200 mg, 0.487 mmol, 1.0 eq.), (3-nitrophenyl)boronic acid (98 mg, 0.585 mmol, 1.2 eq.), tripotassium phosphate (175 mg, 0.829 mmol, 1.7 eq.).) in dioxane (3.0 ml) and water (0.3 ml) was purged with nitrogen for 15 min. Triphenylphosphine (3 mg, 0.011 mmol, 2 mol%) and tris(dibenzylideneacetone)dipalladium (4 mg, 0.005 mmol, 1 mol%) was added and purging was continued for another 10 min. The resulting reaction mixture was heated *via* microwave irradiation to 100 °C for 1.5 h. The reaction mixture was diluted with water (10 ml) and extracted with ethyl acetate (2 × 20 ml). The combined organic layer was washed with brine (1 × 20 ml) and dried over magnesium sulfate. The organic layer was concentrated under vacuum to give a residue which was purified by silica gel column chromatography using ethyl acetate-hexane (0 %–40 %) to afford **12** as an off-white solid (90 mg, 0.197 mmol, 40 %).

#### 2.1.3 Synthesis of 3-((4-chloro-3-nitrophenyl)amino)-4-ethoxycyclobut-3-ene-1,2-dione (**26**)

A solution of 4-chloro-3-nitroaniline (500 mg, 2.897 mmol, 1.0 eq.) and **25** (0.4 ml, 2.897 mmol, 1.0 eq.) in ethanol (10 ml) was stirred at room temperature for 72 h. After completion, the reaction mixture was concentrated under vacuum and the residue was purified by silica gel column chromatography using methanol-dichloromethane (0 %–1 %) to afford 192 as a yellow solid (526 mg, 1.773 mmol, 61 %). Data for this compound were consistent with those reported previously in the literature (Thiele et al., 2022).

#### 2.1.4 Synthesis of 2-(3-bromophenyl)propan-2-amine (**27**)

To a solution of 3-bromobenzonitrile (600 mg, 3.296 mmol, 1.0 eq.) in diethyl ether (10 ml), under nitrogen, methyl magnesium bromide (3M, 3.2 ml, 9.890 mmol, 3.0 eq.) was added and the reaction mixture was stirred at room temperature for 30 min. Titanium isopropoxide (0.90 ml, 3.296 mmol, 1.0 eq.) was slowly added and the reaction mixture was heated to reflux at 80 °C for 18 h. After completion, the reaction mixture was cooled to 0 °C and 2N sodium hydroxide (25 ml) was slowly added. The resulting mixture was allowed to stir at room temperature for 30 min. A saturated solution of sodium bicarbonate (25 ml) was added and the reaction mixture was extracted with ethyl acetate (2 × 30 ml). The organic layer was concentrated under vacuum and the resulting residue was dissolved in 1N hydrochloric acid (10 ml) and extracted with diethyl ether (2 × 30 ml). The aqueous layer was basified (pH 10) by using 2N sodium hydroxide and extracted with ethyl acetate (2 × 25 ml). The combined organic layer was washed with brine (1 × 20 ml) and dried over magnesium sulfate. The organic layer was concentrated under vacuum to afford **27** as a colourless oil (570 mg, 2.662 mmol, 81 %). Data for this compound were consistent with those reported previously in the literature (Hom et al., 2005; Miller, 2016).

#### 2.1.5 Synthesis of 3-((2-(3-bromophenyl)propan-2-yl)amino)-4-((4-chloro-3-nitrophenyl)amino)cyclobut-3-ene-1,2-dione (**28**)

A solution of **27** (100 mg, 0.467 mmol, 1.0 eq.), **26** (165 mg, 0.560 mmol, 1.2 eq.) and tetramethyl guanidine (0.12 ml, 0.934 mmol, 2.0 eq.) in acetonitrile (5 ml) was heated *via* microwave irradiation to 100 °C for 2 h. After completion, the reaction mixture was diluted with water (20 ml) and extracted with ethyl acetate (2 × 25 ml). The combined organic layer was washed with brine (1 × 20 ml) and dried over magnesium sulfate. The organic layer was concentrated under vacuum to afford a residue. The residue was purified by silica gel column chromatography using ethyl acetate-hexane (0 %–40 %) to afford **28** as a yellow solid (178 mg, 0.383 mmol, 82 %).

#### 2.1.6 Synthesis of 3-((2-(3-acetylphenyl)propan-2-yl)amino)-4-((4-chloro-3-nitrophenyl)amino)cyclobut-3-ene-1,2-dione (**29**)

A solution of **28** (100 mg, 0.215 mmol, 1.0 eq.) in dry tetrahydrofuran (3 ml) was cooled to −78 °C and *n*-BuLi (2.5M, 0.1 ml, 0.258 mmol, 1.2 eq.) was slowly added. The reaction mixture was stirred at the same temperature for 30 min. Acetyl chloride (0.05 ml, 0.322 mmol, 1.5 eq.) was added and the reaction mixture was allowed to reach the room temperature over 4 h. After completion, the reaction was carefully quenched with saturated ammonium chloride (10 ml). The reaction mixture was diluted with water (10 ml) and extracted with ethyl acetate (2 × 25 ml). The combined organic layer was washed with brine (1 × 20 ml) and dried over magnesium sulfate. The organic layer was concentrated under vacuum to afford a residue. The residue was purified by silica gel column chromatography using ethyl acetate-hexane (0 %–25 %) to afford **29** as an off-white solid (51 mg, 0.119 mmol, 55 %).

#### 2.1.7 General procedure for the synthesis of substituted squaramides (**30**–**32**)

A solution of **27** (1.0 eq.), the appropriate boronic acid/boronic ester (1.2 eq.), and sodium carbonate (3.0 eq.) in acetonitrile (5 ml) and water (0.5 ml) was purged with nitrogen for 15 min. Tetrakis(triphenylphosphine)palladium (6 mol%) was added and purging was continued for another 10 min. The resulting reaction mixture was heated *via* microwave irradiation to 100 °C for 1.5 h. After completion, the solvent was concentrated under vacuum and the resulting residue was purified by silica gel column chromatography using the stated eluent system.

#### 2.1.8 3-((2-(3-(1H-Pyrazol-3-yl)phenyl)propan-2-yl)amino)-4-((4-chloro-3-nitrophenyl)amino)cyclobut-3-ene-1,2-dione (**30**)

Prepared following the general procedure using **28** (45 mg, 0.096 mmol, 1.0 eq.), 3-(4,4,5,5-tetramethyl-1,3,2-dioxaborolan-2-yl)-1*H*-pyrazole (22 mg, 0.116 mmol, 1.2 eq.), sodium carbonate (30 mg, 0.290 mmol, 3.0 eq.) and tetrakis(triphenylphosphine)palladium (7 mg, 0.005 mmol, 6 mol%). The residue was purified by silica gel column chromatography using methanol-dichloromethane (0 %–2 %) to afford **30** as a yellow solid (26 mg, 0.057 mmol, 60 %).

#### 2.1.9 (3-((2-(3-(1H-Pyrazol-4-yl)phenyl)propan-2-yl)amino)-2-((4-chloro-3-nitrophenyl)amino)-4-oxocyclobut-2-en-1-ylidene)oxonium (**31**)

Prepared following the general procedure using **28** (50 mg, 0.107 mmol, 1.0 eq.), 4-(4,4,5,5-tetramethyl-1,3,2-dioxaborolan-2-yl)-1*H*-pyrazole (25 mg, 0.129 mmol, 1.2 eq.), sodium carbonate (34 mg, 0.322 mmol, 3.0 eq.) and tetrakis(triphenylphosphine)palladium (7 mg, 0.006 mmol, 6 mol%). The residue was purified by silica gel column chromatography using methanol-dichloromethane (0 %–2 %) to afford **31** as a yellow solid (25 mg, 0.055 mmol, 52 %).

#### 2.1.10 (2-((4-Chloro-3-nitrophenyl)amino)-3-((2-(3-(furan-2-yl)phenyl)propan-2-yl)amino)-4-oxocyclobut-2-en-1-ylidene)oxonium (**32**)

Prepared following the general procedure using **28** (50 mg, 0.107 mmol, 1.0 eq.), furan-2-ylboronic acid (14 mg, 0.129 mmol, 1.2 eq.), sodium carbonate (34 mg, 0.322 mmol, 3.0 eq.) and tetrakis(triphenylphosphine)palladium (7 mg, 0.006 mmol, 6 mol%). The residue was purified by silica gel column chromatography using ethyl acetate-hexane (0 %–50 %) to afford **31** as a yellow solid (18 mg, 0.039 mmol, 37 %).

### 2.2 Parasitology

#### 2.2.1 Cell culture

HCT-8 cell lines (Human ileocecal adenocarcinoma) were purchased from The European Collection of Authenticated Cell Cultures (ECACC) under catalogue number No 90032006) and maintained with regular subculturing in a growth medium consisting in a RPMI 1640 medium supplemented with 1 mM sodium pyruvate, 2 mM l-glutamine, 10 % (vol/vol) of heat-inactivated fetal calf serum, 100 U/mL of penicillin and 100 μg/mL of streptomycin. Cells were grown in an incubator at 37 °C with 5 % (vol/vol) CO_2_ until monolayers reached 80–90 % confluency. Cells were cultivated in 96-well format plate for molecular analysis and in Lab-Tek™ Chamber Slides for microscopic analysis. 50,000 cells were seeded and incubated during 24 h (until confluency) before infection.

#### 2.2.2 Infection of HCT-8 cells by C. parvum

*C. parvum* oocysts of “Iowa” strain was purchased from Waterborne Inc. and stored in PBS at 4 °C. A ratio of one oocyst per cell was used to infect HCT-8 cell. Oocysts suspension was incubated with 10 % of a solution (vol/vol) containing 0.5 % sodium hypochlorite for 10 min at 4 °C. After a washing step by centrifugation at 1800*g* for 15 min at 4 °C, oocysts were incubated in acidified water (pH 2.4) containing 0.025 % (wt/vol) of trypsin, at 37 °C for 20 min, to trigger excystation (infectivity assay developed by Keegan et al., 2003). After a third centrifugation step at 1800 g for 10 min, oocysts were suspended in a maintenance medium. The maintenance medium consisted of RPMI 1640 medium with 2 mM l-glutamine, 5 mM glucose, 0.5 μM folic acid, 7 μM 4-amino-benzoic acid, 0.1 μM calcium pantothenate, 50 nM ascorbic acid, 2 % (vol/vol) heat inactivated fetal calf serum and 100 U/mL of streptomycin/penicillin. Before oocyst inoculation, the growth medium was switched with maintenance medium. Triggered oocysts were finally added on monolayers and incubated during 48 h at 37 °C in a 5 % (vol/vol) CO_2_ atmosphere.

#### 2.2.3 Compound preparation and cytotoxicity

The compounds (2 mg/mL in DMSO) were diluted to 500 μM and serially diluted in maintenance medium. The cytotoxicity of the compounds was assessed using the CytoTox 96® Non-Radioactive Cytotoxicity Assay (Promega, G1790) in accordance with the manufacturer's protocol.

#### 2.2.4 Fluorescence staining

After aspiration of the supernatant from the Nunc™ Lab-Tek™ Chamber slide wells, cells were rinsed with PBS 1X. A fixation step was then performed using methanol during 8 min at 4 °C. *Cryptosporidium* developmental forms were labelled with a polyclonal IgG antibodies reaction kit tagged with Cy3 (Sporo-Glo™, Waterborne Inc.) and oocysts were labelled with FL-Crypt-a-Glo™, a fluorescein-labelled mouse monoclonal antibody made to oocyst outer wall antigenic sites (epitopes) of *C. parvum*. Antibodies were 1/20th diluted and incubated on cells for 1 h at room temperature. DNA was labelled with Hoechst for 5 min at room temperature. Slides were then coverslipped with a no fading mounting medium (Waterborne Inc.) and observed with a Leica DMi8 microscope. Each dilution was performed in triplicate and, for each well, ten images were taken at 400× magnification. Parasite quantification was carried out automatically using the StarDist neural network, which was specifically trained to detect and quantify *C. parvum* parasites.

#### 2.2.5 Parasitic quantification by molecular method (qPCR)

The COWP gene (GenBank no. AF248743) was amplified by qPCR following the previously described protocol (Guy et al., 2003), and the parasitic concentration was determined using the standard range of *C. parvum* oocyst concentrations. Briefly, serial dilutions from a pure *C. parvum* oocyst suspension were made starting from a stock solution of 5 × 10^7^ oocysts (4 × 10^5^ – 2 × 10^5^ – 1 × 10^5^ – 5 × 10^3^–2.5 × 10^3^–1.25 × 10^3^ oocysts) and the standard curve for quantifying parasites was created to obtain a correlation between Cq values and the Log of concentration.

#### 2.2.6 Sample preparation and DNA extraction

At the end of the experiment, supernatants from the *C. parvum*-infected cell cultures were collected. The cell layers were then washed (1x PBS), detached using trypsin (0.25 %), and centrifuged (10,000×*g*, 10 min). The resulting cell pellet was re-suspended in 150–200 μL of sterile 1x PBS and stored at −20 °C for later DNA extraction. DNA extraction of *C. parvum* was performed using the Purelink™ Microbiome Purification Kit (Invitrogen™) following the manufacturer's protocol “Transport Media and Microbial Culture Samples” (Invitrogen, 2015).

#### 2.2.7 Real-time qPCR

FRePCR reactions were formulated to a volume of 25 μL and comprised of 1.25 μL of primers (10 μM), 0.05 μL probe (100 μM), 12.5 μL Master Mix 2X, LightCycler 480 Probes Master (ref. 04707494001, Roche), 4.95 μL H_2_O nuclease-free and 5 μL of DNA template. Quantitative PCR was run on a CFX96 real time PCR system (BioRad) with an initial step of activation (10 min 95 °C) following by 40 cycles with cycling conditions as follows: 95 °C for 10 s, 60 °C for 1 min. BioRad CFX manager software was used to analyse the data. Samples were considered negative if Cq ≥ 40.

#### 2.2.8 IC_50_ assessment

A common way of defining a 50 % response is to use a mathematical model like the 4-parameter logistic model (4 PL) (Sebaugh, 2011). The 4 PL model typically resolves as a sigmoid function, or "S"-shaped curve. The formula for the 4 PL may be expressed as:

$$f\left(x\right)=bottom+\frac{top-bottom}{1+{\left(\frac{x}{{IC}_{50}}\right)}^{Hill\;slope}}$$

where “top” is the maximal value; “bottom” is the minimal value; “IC_50_” is the median value and “Hill slope” is the slope of the curve.

#### 2.2.9 Statistical analysis

Statistical analyses were done by two-way analysis of variance (ANOVA) with the Tukey's multiple comparison *post hoc* test. *P*-values of 0.05 or less were considered significant.

## 3 Results

### 3.1 Chemistry

**Fig. 2:**
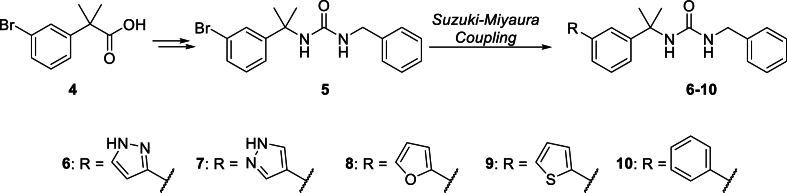
Preparation of nitrophenylurea analogues **6**–**10**.

A series of benzyl-derived urea analogues were prepared as previously outlined (Fig. 2) (Ayoub et al., 2024b). Carboxylic acid **4** was transformed into urea **5** by initial conversion to the carboxylic azide followed by Curtius rearrangement to the isocyanate and then treatment with benzylamine to provide the target. Aryl bromide **5** served as a common intermediate from which aryl/heteroaryl-containing analogues **6**–**10** could be accessed *via* Suzuki-Miyaura coupling with the appropriate boronic acid or ester.

**Fig. 3:**
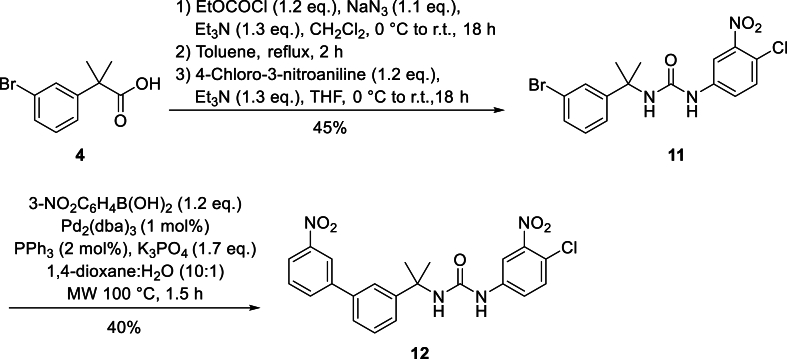
Synthesis of novel nitrophenylurea analogue **12**.

A similar strategy was employed for the preparation of novel nitrophenylurea analogue **12** (Fig. 3). Starting from known carboxylic acid **4**, treatment with ethyl chloroformate and sodium azide afforded the corresponding carboxylic azide. Heating to reflux in toluene initiated a Curtius rearrangement to the isocyanate which was then treated with 4-chloro-3-nitroaniline to provide **11** in 45 % overall yield from **4**. Coupling of **11** using tris(dibenzylideneacetone)dipalladium, triphenylphosphine and tripotassium phosphate in dioxane/water under microwave irradiation afforded **12** in 40 % yield. The low yield was primarily due to interference by the urea functional group with the catalytic cycle, leading to incomplete consumption of starting material, even with extended reaction times.

**Fig. 4:**
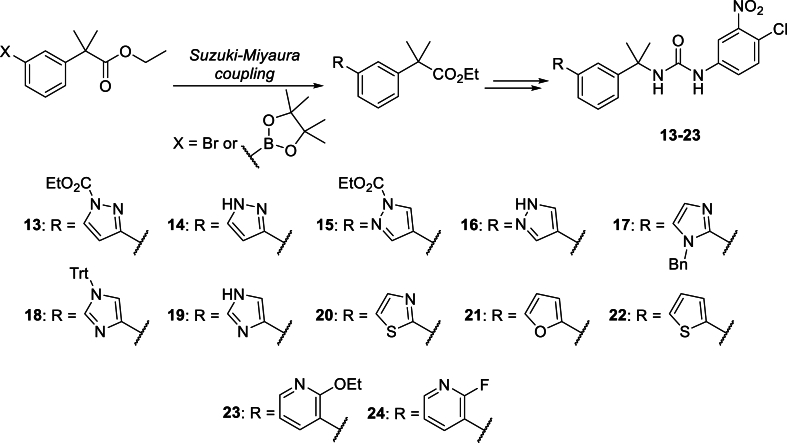
Preparation of urea-based analogues **13**–**24**.

The remaining chloronitrophenyl-substituted ureas were obtained using a modified synthetic approach, whereby Suzuki-Miyaura introduction of the heteroaromatic ring was conducted an earlier stage in order to avoid complications from the urea functional group (Fig. 4) (Ayoub et al., 2024b). This series comprised of twelve analogues, containing a variety of 5-membered (**13**–**22**) and 6-membered (**23**–**24**) heteroaryl substituents.

**Fig. 5:**
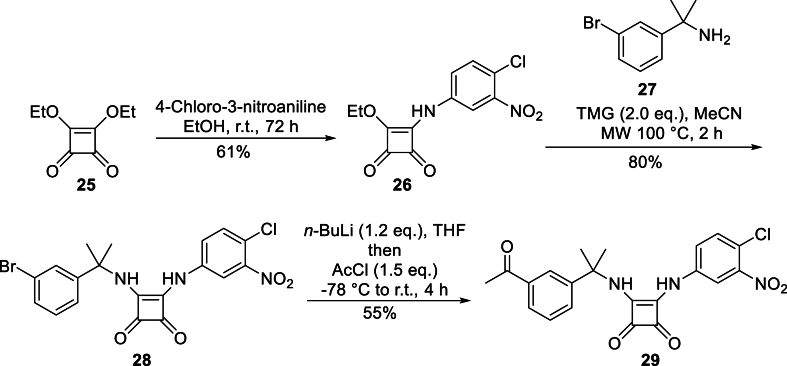
Synthesis of advanced intermediate **28** and ketone **29**.

The strategy of replacing a urea with a squaramide can enhance the therapeutic activity, metabolic stability and aqueous solubility of medicinal compounds (Agnew-Francis and Williams, 2020). Synthesis of our squaramide analogues began with condensation of 4-chloro-3-nitrobenzene with diethyl squarate (**25**) to furnish alkoxy amino squarate **26** in 61 % yield (Fig. 5). The next step involved coupling of **26** with arylamine **27**, which was obtained by reaction of 3-bromobenzonitrile with methylmagnesium bromide in the presence of titanium isopropoxide (Fig. 5) (Miller, 2016). Using a methodology for the coupling of adenosine derivatives with alkoxy amino squarates reported by Zhang (Zhang et al., 2018), stirring of **26** and **27** in the presence of *N*,*N*-diisopropylethylamine provided target squaramide **28**, albeit in a low yield of 20 %. Increasing the equivalents of base and heating under microwave radiation returned an increased yield of 48 % along with significant amounts of unreacted starting material. A subsequent base screen of TMG (p*K*_*a*MeCN_ 23.7), DBU (p*K*_*a*MeCN_ 24.3), and TBD (p*K*_*a*MeCN_ 26.2) saw the best results recorded in the presence of TMG, with an 80 % yield of **28** obtained after heating to 100 °C after 2 h. The formation of **28** was confirmed by the appearance of the squaramide protons as 1H singlets at 7.63 ppm and 10.16 ppm respectively in the ^1^H-NMR spectrum, with the difference in chemical shifts likely due to the electron withdrawing effect of the 4-chloro-3-nitrophenyl ring. In addition to advanced intermediate **28**, ketone **29** was also synthesised by treating **28** with *n*-butyl lithium followed by addition of acetyl chloride to produce **29** in 55 % yield.

A number of heteroaryl groups were coupled to aryl bromide **28** using different boronic acids and esters while employing tetrakis(triphenylphosphine)palladium as the catalyst and sodium carbonate as base in 10:1 acetonitrile/water under microwave irradiation (Table 1). The selected rings included a 3-pyrazole (entry 1) as well as its 4-pyrazole isomer (entry 2) as these motifs had been previously associated with potent inhibition of bacterial IMPDH (Ayoub et al., 2024b). An oxygen-containing furan (entry 3) was also included. The more polar 3-pyrazole and 4-pyrazole rings were coupled as their boronate esters (entries 1 and 2) whereas the 2-furyl was coupled as its boronic acid (entry 3). The Suzuki-Miyaura couplings proved less problematic in the presence of the squaramide group, with yields ranging from 37 % for the furyl substituent (entry 3) to 60 % for the 3-pyrazole ring (entry 1). The reduced yields were mostly due to difficulties in purification rather than incomplete conversions as observed with the urea substrates.

**Table 1:**
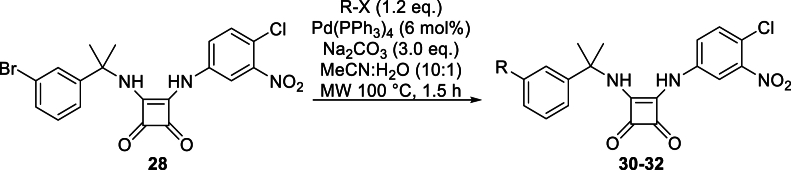
Suzuki-Miyaura couplings of squaramide intermediate **28**.

Entry	R	X	Product	Yield	
1.			**30**	60 %	
2.			**31**	52 %	
3.		-B(OH)_2_	**32**	37 %	

### 3.2 Biological evaluation

**Fig. 6:**
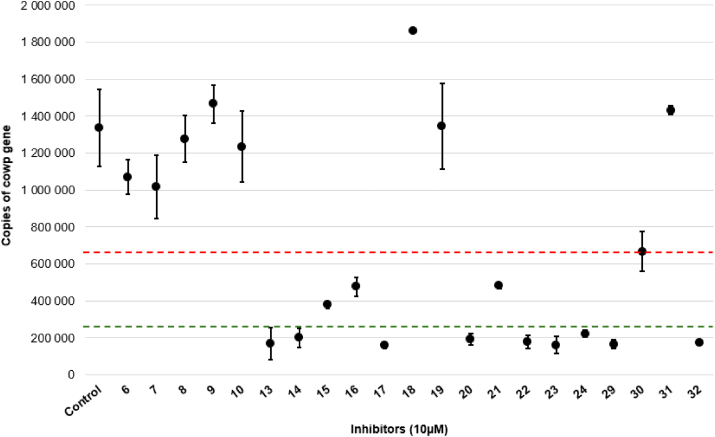
Plot of the anti-*Cryptosporidium* activity of 21 compounds. All compounds were screened at 10 μM. A cut-off of 50 % (dotted red line) and 80 % (dotted green line) inhibition of *C. parvum* was applied. Mean ± SD (n = 3 wells).

An initial screening experiment was conducted on the full library to evaluate the cytotoxicity effects on HCT-8 cells. Nitrophenylurea **12** was identified as being cytotoxic so was not progressed further (*See* Supporting *Information*). In total, 21 candidates were assessed for their ability to inhibit *C. parvum* replication at a concentration of 10 μM. Each compound was added at the start of the infection of HCT-8 cells (T0) and remained in the culture for 48 h post infection (hpi). Parasitic quantification was then performed using a molecular method (qPCR). Of the 21 compounds tested, nine were effective inhibitors, reducing parasite proliferation by 80 % or more (Fig. 6). A clear trend emerged with all of the benzyl-substituted ureas **6–10** displaying poor inhibitory activity, regardless of the attached heteroaryl substituent. By contrast, most of the chloronitrophenyl-substituted analogues **13–24** inhibited *C. parvum* growth by more than 50 %, with greater than 80 % inhibition recorded for several candidates. Two exceptions to this were imidazole-containing analogues **18** and **19**. These findings suggest that the presence of the chloronitrophenyl motif is strongly associated with inhibitory activity (e.g. **13–24**), but the nature of the heteroaryl group is also an important consideration, as demonstrated in the case of **18** and **19**. Among the squaramide series, the results were highly dependent on the nature of the substituent, ranging from high (e.g. ketone **29**, furan **32**), medium (e.g. 3-pyrazole **30**) to low (e.g. 4-pyrazole **31**) anti-cryptosporidial activity.

To confirm our initial findings, we employed an alternative method using immunofluorescence microscopy to quantify *C. parvum* development in the presence of inhibitors (Fig. S1). Validation focused on the most potent urea analogues (**17**–**22**) and the squaramide series (**29**–**32**). The results corroborated our initial screening data, confirming the high efficacy of all tested chloronitrophenyl urea analogues, except for imidazole derivatives **18** and **19**.

We next focused on molecules compounds with an inhibition rate greater than 50 %, namely **13** (87.5 ± 6.4 %), **14** (85.1 ± 3.8 %), **15** (71.7 ± 1.5 %), **16** (64.3 ± 3.9 %), **17** (88.2 ± 1.1 %) **20** (85.7 ± 2.2 %), **21** (64.0 ± 1 %), **22** (86.6 ± 2.6 %), **23** (88 ± 3.5 %), **24** (83.4 ± 1.5 %), **29** (87.7 ± 1.7 %) and **32** (87.2 ± 0.8 %).

The selected molecules were tested on the *C. parvum* parasite across a range of increasing concentrations (i.e. 0, 3, 6 and 9 μM) in order to determine the individual IC_50_ values of the selected molecules. To achieve this, the compounds were added at the start of the infection (t0) and left until 48 h post infection. At the end of the experiment, the samples were collected to quantify the parasite's development using qPCR. The results are presented in Table 2. Of the 12 candidates assayed, the lowest IC_50_ value was recorded for furyl-containing squaramide **32** (entry 12). Ketone-containing squaramide **29** was almost as effective (entry 11), demonstrating that the squaramide group can act as an effective bioisosteric replacement for the urea functional group. Among the urea derivatives, the IC_50_ values ranged from 3.23 μM (entry 9) to 6.19 μM (entry 2) demonstrating good activity across the series. The two most effective urea-based inhibitors both contained a pyridine ring, namely 2-ethoxypyridine **23** (entry 9) and 2-fluoropyridine **24** (entry 10). In the case of the 3- and 4-pyrazoles (entries 2 and 4 respectively), their corresponding *N*-carboxy esters (entries 1 and 3 respectively) were found to be equipotent, facilitating the development of prodrug analogues.

**Table 2:** IC_50_ values of the most active compounds.

Entry	Compound	IC_50_ (μM)	Exp. error (μM)
1.	**13**	5.44	±1.28
2.	**14**	6.19	±0.93
3.	**15**	5.98	±1.04
4.	**16**	5.32	±0.12
5.	**17**	4.69	±0.20
6.	**20**	4.51	±0.12
7.	**21**	6.19	±1.32
8.	**22**	3.77	±0.27
9.	**23**	3.23	±0.60
10.	**24**	3.64	±0.52
11.	**29**	3.59	±0.58
12.	**32**	2.20	±0.86

Previous experimentation based on qPCR and using Paromomycin as a standard of *Cryptosporidium* inhibitor in *in vitro* assays showed a mean IC_50_ of 511 μM. Consequently, our IMPDH inhibitors exhibited IC_50_ values 83 to 223 times lower than the IC_50_ exhibited by Paromomycin.

**Fig. 7:**
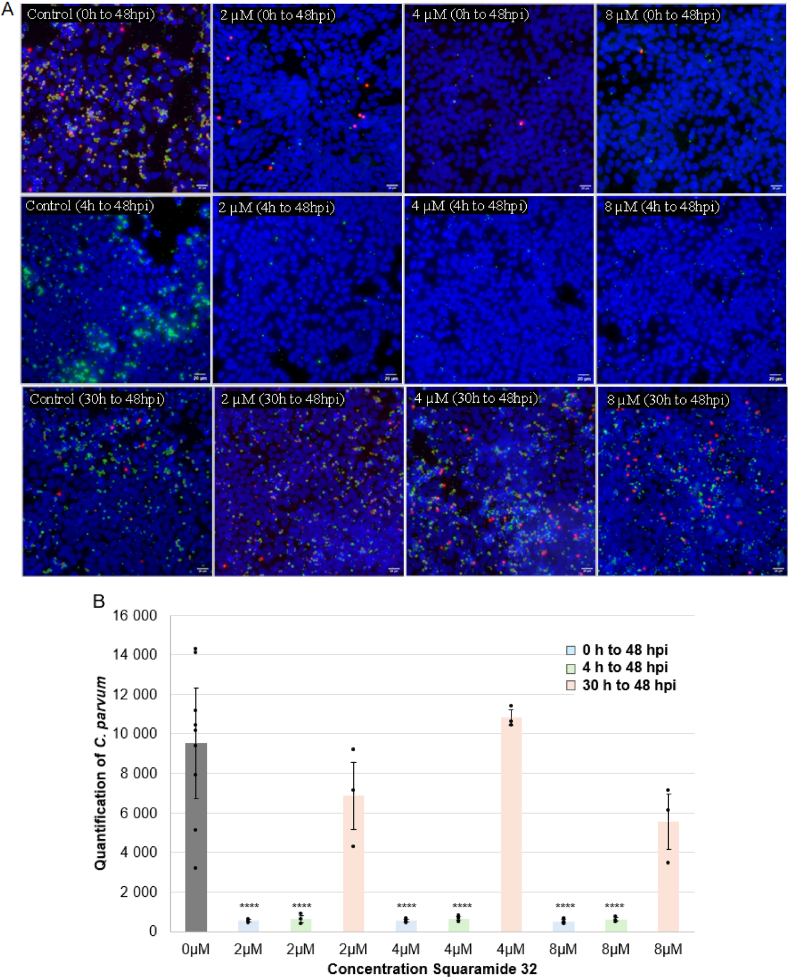
Effect of squaramide **32** at different concentrations on *C. parvum* at 0 hpi, 4hpi and 30hpi. **(A)** Fluorescence microscopy images showing the intracellular development of *C. parvum* (SporoFlo-FL antibody); oocysts in red (Crypt-a-GloCy3 antibody) and nuclei in blue (Hoechst). Magnification x400; scale bar = 20 μm. **(B)** Plot of the anti-*Cryptosporidium* activity at different concentrations of squaramide **32** added at 0hpi (blue), 4hpi (green) and 30 hpi (orange). Mean ± SD (n = 3 wells, ten images per well). ANOVA Tukey's post hoc ∗∗∗∗p ≤ 0.001.

Given that the lowest IC_50_ value was observed with squaramide **32**, additional analyses to determine whether its inhibitory effect was time-dependent or specifically targeted at particular phases of the parasite's life stages were undertaken. To test this hypothesis, increasing concentrations of squaramide **32** (i.e. 0, 2, 4 and 8 μM) were applied at different infection times: at 0 hpi (hours post infection), to assess the impact on parasite invasion; at 4 hpi, to examine the effect on the early stages of intracellular development; and at 30 hpi, to evaluate the influence on late stage intracellular development. The microscopic images in Fig. 7 illustrate the effect of **32** when applied at the onset of infection and at 30 hpi. Using fluorescent antibodies, the intracellular development of *C. parvum* is highlighted by green fluorescence, which allows for quantification of the number of parasites per image. The inhibitory effect of **32** is clearly evident, as even the lowest concentration (2 μM) significantly reduces green fluorescence, indicating suppressed intracellular development. A similar inhibitory effect (Fig. 7A) was observed when **32** was applied at 4 hpi, targeting the early stages of parasite development.

By contrast, the efficacy of compound **32** diminished significantly when administered at 30 hpi, suggesting that its inhibitory effect weakens during the later stages of the parasite's life cycle (Table 2) or with a reduced duration of application (18 h). By this stage, *C. parvum* is firmly established within host cells and may depend less on *de novo* nucleotide biosynthesis for its metabolic requirements. Furthermore, the parasite may utilise alternative strategies, such as host-derived metabolism or nucleotide salvage pathways, reducing its susceptibility to squaramide **32**.

**Fig. 8:**
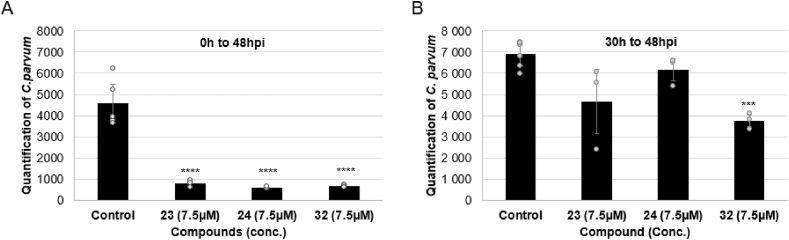
Quantification of *C. parvum* by fluorescent microscopy in presence of **23**, **24** and **32** at 7.5 μM with inhibitors added at 0hpi (A) or (B) 30hpi. Ten images per well (n = 3). Mean ± SD. ANOVA Tukey's post hoc analysis ∗∗∗p ≤ 0.005 ∗∗∗∗p ≤ 0.001.

Previous results had highlighted the loss of efficacy of squaramide **32** when administrated at 30 hpi followed by 18h of treatment. A concentration of 8 μM (>3.5 IC_50_) had reduced the parasite load less by 50 % (Fig. 7B) and this result was confirmed by testing at 7.5 μM which yielded a 46 % reduction, significantly lower than the >80 % reduction observed when **32** was added at 0 hpi (Fig. 8). To ascertain whether this phenomenon was unique to squaramide **32**, the impact of the most effective urea analogues, namely **23** and **24**, on *C. parvum* development was also investigated. At 30 hpi, treatment with compounds **23** and **24** at 7.5 μM (>2 IC_50_) caused a minimal, non-significant reduction in parasite quantifications (32 % and 10.5 % respectively) compared to the control, suggesting that administration of these compounds at this concentration does not significantly inhibit parasite development. Although 2-fluoropyridyl-substituted urea **24** could potentially act as a covalent inhibitor, the reduction in parasite signal at 7.5 μM was smaller when compared to **23**.

**Fig. 9:**
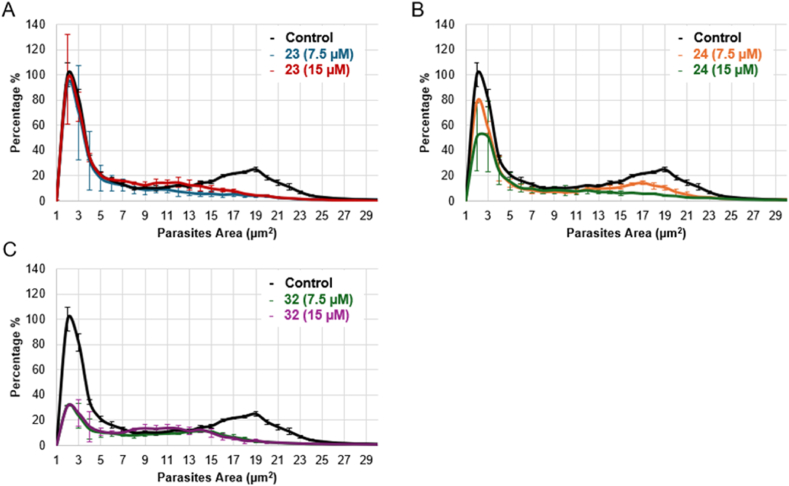
Area of each parasite with or without inhibitors **23** (A), **24** (B) and **32** (C) at 7.5 μM or 15 μM after 30 hpi until 48hpi. Mean ± SD (n = 3 wells).

In light of the quantification data, which demonstrated a loss of efficacy of the compounds when administrated at 30 hpi, further analyses to examine their impact on the distribution of parasite sizes, an indirect measure of *C. parvum* growth and development, were undertaken. Although analogues **23**, **24**, and **32** all potentially target the *Cp*IMPDH enzyme, they do not exhibit the same impact on parasite populations discriminated by their area. Fig. 9 illustrates the distribution of parasite area following treatment with 7.5 μM or 15 μM of these inhibitors. 2-Ethoxypyridyl-substituted urea **23** effectively inhibits parasites, but doubling the concentration is not correlated with an increase of the inhibitory effect. Moreover, while **23** successfully inhibits larger parasites (ranging from 16 μm^2^ to 22 μm^2^), it fails to significantly impact smaller ones (ranging from 1 to 4 μm^2^). By contrast, 2-fluoropyridyl-susbstituted urea **24** demonstrates a dose-dependent effect, reducing both small and large parasite forms more effectively at higher concentrations. Finally, squaramide **32** decreases the prevalence of both small and large parasites at both tested concentrations (7.5 μM and 15 μM). The lack of a dose-dependent effect for squaramide **32** and 2-ethoxypyridyl-substituted urea **23**, despite their low IC_50_ values, while the 2-fluoropyridyl-substituted urea **24** shows a clear dose-dependent effect on all parasite forms, could be explained by several hypotheses: (1) Inhibitors **23** and **32** could reach a saturation concentration, meaning that increasing the dose to 15 μM does not enhance their efficacy. Moreover, other mechanisms of action could be also considered: (2) Inhibitor **24** may penetrate the host cell more efficiently, (3) target the parasite's IMPDH more effectively, or (4) affect a pathway essential for all stages of parasite's life cycle, preventing compensatory mechanisms.

## 4 Discussion

Recent advances in drug discovery have highlighted the potential of IMPDH inhibitors as a promising strategy for treating infections caused by protozoans and bacteria. A wide variety of potential inhibitors have been synthesised and evaluated for their effects on *Cp*IMPDH, including 1,2,3-triazole derivatives (Maurya et al., 2009; Sharling et al., 2010), benzimidazole derivatives (Kirubakaran et al., 2012), urea derivatives (Gorla et al., 2012), benzoxazole derivatives (Gorla et al., 2013), phthalazinone derivatives (Johnson et al., 2013), a series of benzopyrano[4,3-c]pyrazole derivatives (Sun et al., 2014) and adenosine-derived inhibitors (Shigetomi et al., 2019). Among these, urea P131 has emerged as the most advanced candidate, demonstrating superior efficacy in a mouse model of acute infection (Gorla et al., 2014). Notably, P131 outperformed paromomycin when administered in split doses, with its anti-cryptosporidial activity attributed to its accumulation in intestinal epithelial cells*.* Despite these advances, the therapeutic relevance of targeting IMPDH has been challenged. For example, a CRISPR/Cas9-based study on *C. parvum* revealed that multiple genes involved in nucleotide metabolism, including DHFR-TS and IMPDH, are not essential for parasite survival (Pawlowic et al., 2019). It has been proposed that host-derived purine nucleotides are directly imported as ATP raising questions about the fundamental role of these targets *in vivo*.

The metabolic flexibility of *C. parvum* complicates our understanding of IMPDH's role. A study of metabolic differences between virulent and avirulent strains of *Toxoplasma gondii* suggests a potential association between IMPDH expression and the virulent phenotype. Elevated expression of IMPDH in the virulent strain may reflect its contribution to the increase metabolic demands associated with virulence (Zhou et al., 2017). For example, 1,2,3,-triazole A110 showed potent *in vitro* inhibition of *C. parvum* growth (EC_50_ value of <0.8 μM) and was considered a promising candidate for advancement into mouse models of infection (Sharling et al., 2010). However, A110 failed to exhibit *in vivo* antiparasitic activity. Instead, A110 promoted parasite growth, possibly by disrupting gut microbiota, highlighting the complexities of *in vivo* validation.

As described above, P131 remains one of the most promising *Cp*IMPDH inhibitors discovered to date. However, it is also characterised by the presence of a oxime moiety, a functional group which is prone to metabolic degradation. We previously demonstrated that the oxime group can be replaced with chemically stable heteroaryl rings while maintaining activity against IMPDH in ESKAPEE bacteria. These heteroaryl-containing urea analogues might also be expected to inhibit *Cp*IMPDH. In addition, we have now developed a novel set of molecules where the urea motif is replaced with a bioisosteric squaramide. Squaramides are typically more stable than ureas towards nucleophilic attack and are often superior hydrogen bond donors (Storer et al., 2011). Furthermore, experimental studies have revealed that the strength of hydrogen bonds formed by squaramides is similar to or greater than comparable urea analogues (Tomàs et al., 1996; Quiñonero et al., 2000a, 2000b). Due to the presence of an extra carbonyl group in squaramides, additional hydrogen bond interactions are possible which is not the case with simple ureas.

The results show that *in vivo* inhibition of IMPDH leads to a reduction in the development of *Cryptosporidium* parvum. However, the results of this assay can be radically modified by the protocol parameters. In the present study, we screened 21 potential *Cp*IMPDH inhibitors and identified novel squaramide **32** as the most effective candidate, followed by 2-ethoxypyridyl-substituted urea **23** and 2-fluoropyridyl-substituted urea **24.** These molecules demonstrated significant efficacy when administrated at 0 or 4 hpi. However, their reduced efficacy when applied at 30 hpi, suggest a time-dependent impact, potentially due to two factors: (1) a shorter exposure time, as most *in vitro* trials were carried out following 48 hpi; and (2) the ability of *C. parvum* to activate salvage pathways or alternative enzymes, as suggested by Pawlovick et al. (Pawlowic et al., 2019). Consequently, emerging enzymes or transportation processes to provide guanine from the host cell to the parasite could explain why *Cp*IMPDH inhibitors lose their anti-*Cryptosporidium* activity.

The comparison of *Cp*IMPDH inhibitors with other antiparasitic agents highlights the significance of timing in drug efficacy. For example, BRD7929 (Phe-RS inhibitor), blocks nuclear division and arrest development at the trophozoite, demonstrating potency when administrated early (Funkhouser-Jones et al., 2020). By contrast, KDU691 (PI(4)K inhibitor), is effective at later stages, preventing merozoite egress. These results highlight the importance of understanding the metabolic demands of *C. parvum* across its life-cycle to optimise therapeutic strategies.

Although compounds **23**, **24**, and **32** share a common target in *Cp*IMPDH, our data suggest that they inhibit *C. parvum* development through distinct mechanisms during parasite life-cycle. A recent study has shown that some antiparasitic compounds inhibit all stages, while others specifically target asexual stages proliferation or selectively block macrogamont differentiation and maturation (Hasan et al., 2024). Such effects would not have been detected using the standard asexual growth assay. The authors also note that inhibitors of sexual differentiation may reduce macrogamont numbers without significantly affecting the total parasite count. We observed similar results when **23**, **24** and **32** were added at 30hpi, in that the parasite count did not differ significantly, but the distribution of parasite area was altered. Structural differences between these compounds may affect their binding affinities or binding modes. It is also possible that these inhibitors impact additional, unidentified pathways or processes.

Accordingly, the present study represents a first step in the development of squaramide-based *Cp*IMPDH inhibitors as well as the improvement of *in vitro* drug screening assays. These squaramide derivatives could be also tested for their capacity to inhibit a broader range of protozoan parasite such as *Plasmodium* (Raza et al., 2017), *Eimeria* (Hupe et al., 1986), *Toxoplasma* (Sullivan et al., 2005), *Babesia* (Cao et al., 2013), *Trypanosoma* (Wilson et al., 1994) or *Leishmania* (Wilson et al., 1991).

## 5 Conclusion

The limited range of effective treatments for Cryptosporidiosis in both humans and animals highlights the urgent need to develop novel therapeutics. Many previous approaches targeted major metabolic pathways, including nucleotide synthesis mediated by *Cp*IMPDH. In this present work, we have developed and evaluated novel IMPDH inhibitors and assessed their impact on *Cryptosporidium* growth *in vitro*. Our results highlight also the need for a commonly accepted standard protocol for *in vitro* trials of anti-cryptosporidial compounds.

## CRediT authorship contribution statement

**Anne-Charlotte Lenière:** Writing – original draft, Methodology, Investigation, Formal analysis, Data curation. **Amit Upadhyay:** Writing – original draft, Methodology, Investigation, Data curation. **Jérôme Follet:** Writing – review & editing, Writing – original draft, Supervision, Resources, Funding acquisition, Conceptualization. **Timothy P. O'Sullivan:** Writing – review & editing, Writing – original draft, Supervision, Funding acquisition, Conceptualization.

## Declaration of interests

The authors declare the following financial interests/personal relationships which may be considered as potential competing interests: Amit Upadhyay reports financial support was provided by 10.13039/501100002081Taighde Éireann – Research Ireland. If there are other authors, they declare that they have no known competing financial interests or personal relationships that could have appeared to influence the work reported in this paper.
