# Supplementary material for: Effect of urea and squaramide IMPDH inhibitors on C. parvum: in vitro trial design impacts the assessment of drug efficacy
Source: Int J Parasitol Drugs Drug Resist. 2025 Apr 15;28:100592. doi: 10.1016/j.ijpddr.2025.100592 (PMC12123366; doi:10.1016/j.ijpddr.2025.100592)
Supplement: Multimedia component 1 [file mmc1.docx]

**Supplementary data**

**Table S1.** Cytotoxicity assessment of compounds at 10 µM. Value of viability +/- SD.

| **Compound** | **Viability**  **(%)** | **Exp. error**  **(%)** |
| --- | --- | --- |
| **6** | 97.04 | ±0.33 |
| **7** | 96.73 | ±0.89 |
| **8** | 95.87 | ±1.05 |
| **9** | 95.39 | ±1.36 |
| **10** | 97.44 | ±0.39 |
| **12** | 57.99 | ±19 |
| **13** | 97.16 | ±0.13 |
| **14** | 97.57 | ±0.43 |
| **15** | 97.50 | ±0.38 |
| **16** | 96.43 | ±1.20 |
| **17** | 97.32 | ±1.05 |
| **18** | 97.34 | ±0.74 |
| **19** | 97.41 | ±0.63 |
| **20** | 98.39 | ±0.21 |
| **21** | 98.31 | ±0.20 |
| **22** | 98.45 | ±0.11 |
| **23** | 94.08 | ±1.05 |
| **24** | 96.80 | ±1.85 |
| **29** | 97.18 | ±0.76 |
| **30** | 98.20 | ±0.07 |
| **31** | 96.97 | ±0.98 |
| **32** | 94.94 | ±2.81 |


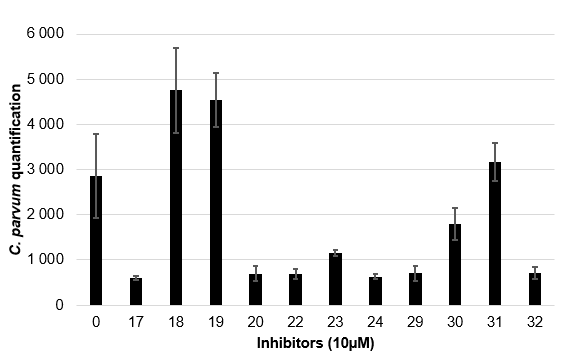


**Fig. S1.** Plot of the anti-*Cryptosporidium* activity of the full compound library. All compounds were screened at 10 µM. A cut-off of 50% (dotted red line) and 80% (dotted green line) inhibition of *C. parvum* was applied. Mean ± SD (n= 3 wells).

**Spectroscopic Data**

*1-(2-(3-Bromophenyl)propan-2-yl)-3-(4-chloro-3-nitrophenyl)urea (****11****)*

**^1^H NMR** (500 MHz, CD_3_OD): δ 1.67 (6H, s, H7), 7.24 (1H, app. t, *J* = 7.8 Hz, H3), 7.37 (1H, ddd, *J* = 7.9 Hz, 1.8 Hz, 0.9 Hz, H4), 7.42-7.46 (3H, m, Ar**H**), 7.48 (1H, app. t, *J* = 1.8 Hz, H6), 7.92 (1H, d, *J* = 2.1 Hz, H17).

**^13^C NMR** (75 MHz, DMSO-*d_6_*): δ 29.8 (C7), 54.7 (C8), 113.7 (C17), 116.2 (Ar**C**), 122.0 (C1), 122.7 (Ar**C**), 124.4 (C15), 128.1 (Ar**C**), 129.3 (Ar**C**), 130.7 (Ar**C**), 132.0 (Ar**C**), 140.8 (C12), 147.9 (C16), 151.4 (C5), 153.9 (C10).

**IR (ATR, cm^-1^)**: 3333, 3103, 2924, 2853, 1648, 1550, 1536, 1362, 821, 694.

**HRMS** (ESI^+^): Exact mass calculated for C_16_H_16_^79^Br^35^ClN_3_O_3_^+^ (M+H^+^) 412.0058. Found: 412.0053.

**Melting point**: 190-192 °C.

*1-(4-Chloro-3-nitrophenyl)-3-(2-(3'-nitro-[1,1'-biphenyl]-3-yl)propan-2-yl)urea (****12****)*

**^1^H NMR** (600 MHz, Acetone*-d_6_*): δ 1.77 (6H, s, H7), 6.65 (1H, s, H9), 7.45-7.60 (5H, m, Ar**H**), 7.73 (1H, app. t, *J* = 7.9 Hz, H22), 7.87 (1H, s, H11), 8.09 (1H, d, *J* = 7.6 Hz, H23), 8.21 (1H, d, *J* = 7.7 Hz, H21), 8.27 (1H, d, *J* = 2.5 Hz, H13), 8.44 (1H, app. t, *J* = 1.9 Hz, H6), 8.60 (1H, s, H19).

**^13^C NMR** (150 MHz, Acetone-*d_6_*): δ 30.0 (C7), 56.0 (C8), 114.6 (C13), 117.3 (Ar**C**), 122.3 (Ar**C**), 122.7 (Ar**C**), 122.9 (Ar**C**), 124.7 (C15), 125.9 (Ar**C**), 126.1 (Ar**C**), 129.8 (Ar**C**), 131.0 (Ar**C**), 132.3 (Ar**C**), 134.1 (C12), 139.2 (Ar**C**), 141.6 (Ar**C**), 141.7 (Ar**C**), 143.9 (C1), 149.0 (C5), 149.7 (C14), 150.2 (C20), 154.4 (C10).

**IR** (ATR, cm^-1^): 3344, 2922, 2855, 1656, 1530, 1348, 1247, 739.

**HRMS** (ESI^+^): Exact mass calculated for C_22_H_20_^35^ClN_4_O_5_^+^ (M+H^+^) 455.1117. Found: 455.1115.

**Melting Point**: 90-93 °C.

*3-((4-Chloro-3-nitrophenyl)amino)-4-ethoxycyclobut-3-ene-1,2-dione (****26****)*

**^1^H NMR** (500 MHz, DMSO-*d_6_*): δ 1.43 (3H, t, *J* = 7.1 Hz, H13), 4.78 (2H, q, *J* = 6.9 Hz, H12), 7.64 (1H, dd, *J* = 8.7 Hz, 2.5 Hz, H11), 7.75 (1H, d, *J* = 8.7 Hz, H10), 8.09 (1H, d, *J* = 2.4 Hz, H7), 11.10 (1H, br s, H5).

**^13^C NMR** (125 MHz, DMSO-*d_6_*): δ 15.9 (C13), 70.5 (C12), 116.4 (C7), 119.5 (C9), 124.7 (C11), 132.8 (C10), 138.7 (C6), 147.9 (C8), 169.6 (C1), 179.7 (C4), 184.7 (C2), 187.8 (C3).

**HRMS** (ESI^+^): Exact mass calculated for C_12_H_10_^35^ClN_2_O_5_^+^ (M+H^+^) 297.0273. Found: 297.0272.

*2-(3-Bromophenyl)propan-2-amine (****27****)*

**^1^H NMR** (300 MHz, CDCl_3_): δ 1.41 (6H, s, H7), 1.83 (2H, br s, H9), 7.12 (1H, app. t, *J* = 7.8 Hz, H3), 7.27 (1H, ddd, *J* = 7.8 Hz, 1.7 Hz, 0.9 Hz, H4), 7.35 (1H, ddd, *J* = 7.7 Hz, 1.6 Hz, 0.9 Hz, H2), 7.59 (1H, app. t, *J* = 1.8 Hz, H6).

**^13^C NMR** (125 MHz, CDCl_3_): δ 32.8 (C7), 52.3 (C8), 122.5 (C1), 123.4 (C4), 128.1 (C6), 129.2 (C2), 129.7 (C3), 152.8 (C5).

*3-((2-(3-Bromophenyl)propan-2-yl)amino)-4-((4-chloro-3-nitrophenyl)amino)cyclobut-3-ene-1,2-dione (****28****)*

**^1^H NMR** (400 MHz, DMSO-*d_6_*): δ 1.79 (6H, s, H13), 7.34 (1H, app. t, *J* = 7.6 Hz, H17), 7.45-7.49 (2H, m, Ar**H**), 7.63 (1H, s, H12), 7.69-7.75 (2H, m, Ar**H**), 8.21 (1H, d, *J* = 1.9 Hz, H7), 8.34 (1H, s, H20), 10.16 (1H, s, H5).

**^13^C NMR** (100 MHz, DMSO-*d_6_*): δ 30.3 (C13), 57.9 (C14), 114.6 (Ar**C**), 117.6 (Ar**C**), 121.8 (C9), 122.9 (C19), 124.2 (Ar**C**), 128.0 (Ar**C**), 129.8 (Ar**C**), 130.5 (Ar**C**), 132.4 (Ar**C**), 139.0 (C6), 147.8 (C15), 149.5 (C8), 163.7 (C1), 170.0 (C4), 180.6 (C2), 182.4 (C3).

**IR** (ATR, cm^-1^): 3271, 2924, 2852, 1706, 1601, 1575, 1534, 1473, 1382, 1344, 1245, 735, 695.

**HRMS** (ESI^+^): Exact mass calculated for C_19_H_16_^79^Br^35^ClN_3_O_4_^+^ (M+H^+^) 464.0007. Found: 464.0009.

**Melting Point**: 188-192 °C.

*3-((2-(3-Acetylphenyl)propan-2-yl)amino)-4-((4-chloro-3-nitrophenyl)amino)cyclobut-3-ene-1,2-dione (****29****)*

**^1^H NMR** (500 MHz, CDCl_3_): δ 1.78 (6H, s, H13), 2.01 (3H, s, H22), 7.15-7.19 (2H, m, Ar**H**), 7.31-7.34 (2H, m, Ar**H**), 7.46 (1H, dd, *J* = 8.5 Hz, 2.4 Hz, H11), 7.50 (1H, app. t, *J* = 1.8 Hz, H20), 7.62 (1H, d, *J* = 8.2 Hz, H10), 7.86 (1H, d, *J* = 2.5 Hz, H7), 8.09 (1H, s, H5).

**^13^C NMR** (125 MHz, CDCl_3_): δ 23.5 (C22), 30.9 (C13), 58.8 (C14), 122.8 (C7), 123.7 (C9), 125.6 (Ar**C**), 128.3 (Ar**C**), 129.2 (Ar**C**), 130.3 (Ar**C**), 130.6 (Ar**C**), 132.9 (Ar**C**), 133.4 (C19), 136.8 (C6), 147.8 (C15), 148.6 (C8), 159.6 (C21), 170.3 (C1), 173.5 (C4), 181.0 (C2), 187.1 (C3).

**IR** (ATR, cm^-1^): 3307, 2926, 1788, 1725, 1698, 1589, 1535, 1401, 1367, 1245, 763.

**HRMS** (ESI^+^): Exact mass calculated for C_21_H_19_^35^ClN_3_O_5_^+^ (M+H^+^) 428.1007. Found: 428.1004.

**Melting Point**: 90-94 °C.

*3-((2-(3-(1H-Pyrazol-3-yl)phenyl)propan-2-yl)amino)-4-((4-chloro-3-nitrophenyl)amino)cyclobut-3-ene-1,2-dione (****30****)*

**^1^H NMR** (500 MHz, CD_3_OD): δ 1.94 (6H, s, H13), 6.71 (1H, br s, H22), 7.43-7.50 (2H, m, Ar**H**), 7.57 (1H, d, *J* = 8.9 Hz, H10), 7.65-7.70 (3H, m, Ar**H**), 7.96 (1H, br s, H20), 8.21 (1H, br s, H7).

**^13^C NMR** (150 MHz, DMSO-*d_6_*): δ 30.9 (C13), 58.6 (C14), 102.6 (C22), 114.9 (Ar**C**), 115.0 (Ar**C**), 118.0 (C7), 122.3 (C9), 123.2 (Ar**C**), 123.3 (Ar**C**), 124.4 (Ar**C**), 124.6 (Ar**C**), 129.3 (Ar**C**), 132.9 (C23), 139.5 (C6), 139.6 (C15), 147.6 (C8), 148.3 (C21), 164.0 (C1), 170.6 (C4), 180.9 (C2), 182.8 (C3)

**IR** (ATR, cm^-1^): 3269, 2924, 1788, 1681, 1609, 1573, 1532, 1434, 1409, 1351, 1259, 699.

**HRMS** (ESI^+^): Exact mass calculated for C_22_H_19_^35^ClN_5_O_4_^+^ (M+H^+^) 452.1120. Found: 452.1119.

**Melting Point**: 210-216 °C.

*(3-((2-(3-(1H-Pyrazol-4-yl)phenyl)propan-2-yl)amino)-2-((4-chloro-3-nitrophenyl)amino)-4-oxocyclobut-2-en-1-ylidene)oxonium (****31****)*

**^1^H NMR** (400 MHz, DMSO-*d_6_*): δ 1.84 (6H, s, H13), 7.27 (1H, d, *J* = 7.7 Hz, H16). 7.36 (1H, app. t, *J* = 7.7 Hz, H17), 7.53 (1H, d, *J* = 7.4 Hz, H18), 7.70-7.74 (3H, m, Ar**H**), 7.96 (1H, s, H20), 8.22 (2H, s, H22, H24), 8.36 (1H, s, H7), 10.21 (1H, s, H5), 12.93 (1H, s, H23).

**^13^C NMR** (125 MHz, DMSO-*d_6_*): δ 30.8 (C13), 58.7 (C14), 115.0 (Ar**C**), 118.0 (C7), 121.6 (Ar**C**), 122.5 (C9), 123.0 (C21), 123.3 (Ar**C**), 124.4 (Ar**C**), 129.3 (C22, C24), 132.9 (Ar**C**), 133.5 (Ar**C**), 139.6 (C19), 147.6 (C15), 148.3 (C8), 164.1 (C1), 170.6 (C4), 180.9 (C2), 182.9 (C3).

**IR** (ATR, cm^-1^): 3279, 2921, 2824, 1788, 1665, 1579, 1536, 1456, 1376, 1260, 797.

**HRMS** (ESI^+^): Exact mass calculated for C_22_H_19_^35^ClN_5_O_4_^+^ (M+H^+^) 452.1120. Found: 452.1120.

**Melting Point:** 168-171 °C.

*(2-((4-Chloro-3-nitrophenyl)amino)-3-((2-(3-(furan-2-yl)phenyl)propan-2-yl)amino)-4-oxocyclobut-2-en-1-ylidene)oxonium (****32****)*

**^1^H NMR** (400 MHz, Acetone-*d_6_*): δ 1.93 (6H, s, H13), 6.53-6.55 (1H, m, H23), 6.86 (1H, d, *J* = 3.0 Hz, H22), 7.39-7.48 (2H, m, Ar**H**), 7.60-7.64 (4H, m, Ar**H**), 7.75 (1H, d, *J* = 7.4 Hz, H18), 7.89 (1H, s, H20), 8.20 (1H, s, H7), 9.24 (1H, br s, H5).

**^13^C NMR** (150 MHz, Acetone-*d_6_*): δ 30.1 (C13), 58.4 (C14), 105.6 (C22), 111.8 (C23), 114.6 (Ar**C**) 118.1 (C7), 120.5 (Ar**C**), 122.5 (C9), 122.6 (Ar**C**), 124.3 (Ar**C**), 128.4 (Ar**C**), 129.0 (Ar**C**), 131.1 (C19), 132.3 (Ar**C**), 139.5 (Ar**C**), 142.5 (C24), 147.6 (C15), 148.5 (C8), 153.6 (C21), 163.8 (C1), 170.7 (C4), 181.2 (C2, C3).

**IR** (ATR, cm^-1^): 3274, 2922, 2852, 1789, 1674, 1611, 1572, 1536, 1444, 1367, 1259, 1249, 795.

**HRMS** (ESI^+^): Exact mass calculated for C_23_H_19_^35^ClN_3_O_5_^+^ (M+H^+^) 452.1007. Found: 452.1005.

**Melting Point**: 188-193 °C.
